# Caries status of first-born child is a predictor for caries experience in younger siblings

**DOI:** 10.1007/s00784-021-04003-6

**Published:** 2021-07-01

**Authors:** Andreina Grieshaber, Asin Ahmad Haschemi, Tuomas Waltimo, Michael M. Bornstein, Eva M. Kulik

**Affiliations:** 1 Department of Oral Health & Medicine, University Center for Dental Medicine Basel (UZB), University of Basel, 4058 Basel, Switzerland; 2 Department of General Pediatric and Adolescent Dentistry, University Center for Dental Medicine Basel (UZB), University of Basel, 4058 Basel, Switzerland; 3 Department Research, University Center for Dental Medicine Basel (UZB), University of Basel, 4058 Basel, Switzerland

**Keywords:** Children, Dental caries, DMFT/dmft, Epidemiology, Intrafamilial, Public oral health

## Abstract

**Objectives:**

This study analysed if children of families in need of dental interventions can be identified by using the caries status of the first-born child as a predictor for caries in younger siblings of the same family.

**Material and methods:**

All children aged 4 to 15 years, i.e. 13,596 children, visiting a compulsory school in the canton of Basel-Stadt, Switzerland, during the school year 2017/2018 were analysed. Total caries experience and untreated carious lesions at time of examination were recorded as well as a subset of socioeconomic factors such as gender, age, nationality, birth order and the family’s place of residence.

**Results:**

A total of 6738 schoolchildren who had at least one sibling of school age could be included. Differences in caries experience and the presence of active carious lesions were found for age, nationality and place of residence but not for gender or birth order. Younger siblings had odds of having a history of caries 3.7 times higher (95% confidence interval: 3.0–4.4) and odds of having active carious lesions 3.5 times higher (95% confidence interval: 2.6–4.7) if the eldest child in the family already had caries.

**Conclusion:**

Caries could be shown to be family-dependent. Younger siblings had a more than three-fold higher risk for caries if the first-born child already had carious lesions.

**Clinical relevance:**

Based on these results, the caries status of the first-born child could be used as a potential indicator to detect vulnerable families and to initiate targeted preventive measures.

## Introduction

Dental caries is a multi-factorial and highly prevalent oral disease affecting both children and adults [1, 2]. By the implementation of population-wide, individual preventive measures such as the use of fluoridated dentifrices and toothpastes, the reduction of dietary sugars or school-based intervention programs, a decline in the prevalence of caries could be observed in many industrialised countries. Although largely preventable, dental caries remains a major health problem globally, and its prevalence is still increasing in low- and middle-income countries [3–5].

In Switzerland, caries prevention programs were initiated in the 1960s, and a reduction in dental caries could initially be observed [6–8]. Lately, an increase in caries in the primary dentition was also noticed in Switzerland, which was thought to result mainly from increasing proportions of schoolchildren with a migrant background [6, 7]. In addition to a migrant background, the socioeconomic status, lifestyle factors, parental caring behaviours and caries in a sibling have been described as potential influencing factors for the caries prevalence in children [9–12]. Recent studies started to explore the association between birth order, i.e. the position of a child in the birth sequence of a family, and children’s oral health. However, results are conflicting as an association between birth order and caries could be demonstrated in some of the studies only [13–15].

In the canton of Basel-Stadt, annual dental examinations are mandatory for all children visiting a compulsory school, including nursery schools, creating a comprehensive dataset for the analysis of caries in schoolchildren across the canton. To ensure that resources are utilised carefully and in a targeted manner, interventions and preventive measures should focus mainly on families and their children in need of oral healthcare. Socioeconomic factors are known to play an important role in explaining differences in oral health. This also applies for dental caries where, for example, education or income has been identified as influencing factors [4, 16]. Nevertheless, it is not always possible to obtain all relevant information on demographic characteristics and socioeconomic determinants of any specific child.

The aim of this study was to identify families in need of dental interventions regarding caries treatment in order to provide targeted preventive measures to these families. Therefore, the null hypothesis of this study was that it is not possible to identify susceptible families by using the caries status of the first-born child as a predictor for caries experience in younger siblings.

## Material and methods

### Study population

In the canton of Basel-Stadt, Switzerland, annual dental examinations are mandatory for all children visiting a compulsory school. Compulsory schooling in Switzerland lasts for 11 years, including 2 years of nursery school. Therefore, all children above the age of 4 visiting a compulsory school during the school year 2017/2018, i.e. between August 1, 2017, and July 31, 2018, were included in this retrospective analysis.

Ethical approval was obtained from the Ethics Committee for the Northwest- and Central Switzerland (EKNZ 2016–02024).

### Dental examinations

Teams consisting of ten paediatric dentists and dental assistants of the University Center for Dental Medicine Basel (UZB) conducted the dental examinations. The dentists are employed at the UZB and mainly perform these mandatory examinations on behalf of the canton on a routine basis. All examiners were initially trained in the diagnosis of caries by experienced dentists.

Up to 4th grade, the schoolchildren brushed their teeth under the supervision of trained oral health instructors prior to examinations. The children were then examined in a mobile dental practice. This specially designed facility enables the dentists to perform the examination in a habitual environment for the children and, at the same time, provides a professional dental unit setting with dental examination light. No magnification glasses were used. If needed, teeth were cleaned before examination with a tissue. To confirm the visual diagnosis of caries, a sickle probe (Maillefer Nr. 6, Maillefer, Ballaigues, Switzerland) was used. No bitewings or other X-ray examinations were made for caries diagnostics [17].

Caries data were entered on-site in an electronic database (StomaNet, Asparagus Engineering AG, Andelfingen, Switzerland), whereas the corresponding medical history data of the children were recorded in a separate medical management system (Vitodent, Vitodata AG, Oberohringen, Switzerland).

### Data collection

For analysis, all data were exported in Excel (Microsoft Corporation, Redmont, WA, USA). The DMFT and dmft indexes were used to describe the caries experience with caries lesions requiring treatment, i.e. lesions extending into the dentin (D3,4) only. After calculating the respective dmft and DMFT values, both numbers were added, and the total caries experience was presented as a dichotomous variable: 1 (i.e. dmft + DMFT > 0) representing a caries experience or 0 (i.e. dmft + DMFT = 0) representing a caries-free dentition. To determine the presence or absence of untreated carious lesions, the dt/DT components were used in a similar manner: 1 (i.e. dt + DT > 0) representing the presence of active caries and 0 (i.e. dt + DT = 0) representing the absence of active caries at the time of examination.

Other variables included gender, birth date, date of examination, nationality and postal code of the place of residence as well as the respective dentist who conducted the dental examination. The age at the time of examination was calculated by the difference between the date of examination and the date of birth. To determine siblings of the same family, names, addresses and telephone numbers of the legal guardians were compared and manually coded with a unique number. Schoolchildren with an incomplete dataset were excluded from the study. The data were anonymised prior to the statistical analysis.

### Statistical analysis

Descriptive statistics are presented as counts and frequencies for categorical data and mean (standard deviation, SD) and median (interquartile range, IQR) for metric variables as appropriate. Overall *p *values correspond to T-test (for means), Kruskal–Wallis test (for median) and Chi-squared or exact Fisher test when the expected frequencies are less than 5 in some cell.

To estimate the variability between families, dentists and postal code of the place of residence, generalised linear mixed effects models were performed. Results are reported as quantiles of the respective distributions.

In order to estimate the within family effect of the outcomes 'caries experience' or ‘active caries’ (yes, no), logistic regression was performed. Caries of the first child was predicted from age, nationality and gender. Caries of the second child was predicted from caries of the first child, age, gender, nationality and age difference to the first child. Similar procedures were performed for subsequent children. Age and age difference were modelled as a five-knot restricted cubic spline to detect possible nonlinearities [18]. Results are reported as odds ratios (OR) with 95% confidence intervals (CI 95%) and *p *values. For ordinal or metric predictors, OR are presented comparing the predictor from the first to the third quartile.

A *p *value < 0.05 is considered significant. All evaluations were done using the statistical software R (R Foundation for Statistical Computing, Vienna, Austria, R version 3.6.1.).

## Results

### Demographics of study population and caries indices

During the school year 2017/2018, a total of 13,596 schoolchildren were examined during the mandatory dental examinations. Out of these, 6775 of these children had at least one sibling of school age that was also examined during the same school year. Due to unclear family relationship or because the respective dental examiner could not be properly assigned, 37 children had to be excluded. This resulted in a total of 6738 siblings belonging to 3089 families that could be included in this study. The age range of the schoolchildren was from 3.9 to 17.9 years, with a male (*n* = 3466; 51.4%) to female (*n* = 3272; 48.6%) ratio of 1.06. Approximately two-thirds of the children (*n* = 4242; 63.0%) were of Swiss nationality, while 2496 (37.0%) had a migrant background.

The mean dmft was 1.42 (SD: 2.76) with a range from 0 to 20, whereas the mean DMFT was 0.54 (SD: 1.32) with a range from 0 to 19. The corresponding values for active caries were 0.38 (SD: 1.29) with a range from 0 to 16 for the primary dentition (dt) and 0.13 (SD: 1.29) with a range from 0 to 11 for the permanent dentition (DT). Approximately half of the children (*n* = 3499; 51.9%) had a caries-free dentition (dmft + DMFT = 0). Teeth with active carious lesions, i.e. with a dt + DT value > 0, were present in 1335 children (19.8%).

### Socioeconomic and confounding factors

The presence or absence of caries as well as active carious lesions was not statistically significant different for gender. However, statistically significant differences were found for age (*p* < 0.001) and nationality with schoolchildren of Swiss nationality having a lower total caries experience and less active carious lesions (Table 1).

**Table 1:** Caries experience (dmft + DMFT) and active carious lesions (dt + DT) by gender and nationality. Total numbers and percentages (in brackets) for schoolchildren without or with caries experience (dmft + DMFT = 0 or dmft + DMFT > 0, respectively) or active carious lesions (dt + DT = 0 or dt + DT > 0, respectively)

	All (*n* = 6738)	Caries experience			Active caries		
		No dmft + DMFT = 0 (*n* = 3499)	Yes dmft + DMFT > 0 (*n* = 3239)	*p *Value	No dt + DT = 0 (*n* = 5403)	Yes dt + DT > 0 (*n* = 1335)	*p *Value
Gender							
Male	3466 (51.4%)	1755 (50.2%)	1711 (52.8%)	0.030	2745 (50.8%)	721 (54.0%)	0.039
Female	3272 (48.6%)	1744 (49.8%)	1528 (47.2%)		2658 (49.2%)	614 (46.0%)	
Nationality							
Swiss	4242 (63.0%)	2442 (69.8%)	1800 (55.6%)	< 0.001	3601 (66.6%)	641 (48.0%)	< 0.001
Foreign	2496 (37.0%)	1057 (30.2%)	1439 (44.4%)		1802 (33.4%)	694 (52.0%)	

When analysing only schoolchildren with caries, the risk for caries experience was between 46.5 and 49.9% and between 16.5 and 25.9% for active carious lesions for the different birth orders (Table 2). No association between birth order and caries experience could be detected (*p* = 0.061), whereas more active carious lesions were present in later-born children (*p* < 0.001).

**Table 2:** Schoolchildren of different birth orders with carious lesions. Shown are total numbers and percentages (in brackets) for children with caries experience (dmft + DMFT > 0) or active carious lesions (dt + DT > 0). *p *values were obtained assuming independence between birth order groups

Birth order		Caries experience (dmft + DMFT > 0)		Active caries (dt + DT > 0)	
	N	n (%)	*p *Value	n (%)	*p *Value
All children	6738	3239 (48.1%)	0.061	1335 (19.8%)	< 0.001
First-born	3089	1540 (49.9%)		510 (16.5%)	
Second-born	3089	1436 (46.5%)		681 (22.0%)	
Third-born	494	231 (46.8%)		128 (25.9%)	
Fourth- and later-born	66	32 (48.5%)		16 (24.2%)	

The number of schoolchildren analysed by the ten paediatric dentists varied between 44 (0.7%) and 1287 (19.1%). The interobserver variability, as determined by calculating the 50% IQR, ranged from 0.47 to 0.53 for caries experience and from 0.12 to 0.16 for active caries suggesting good agreement between the examiners. A larger difference was noted between families, i.e. interfamilial, with 50% IQR from 0.33 to 0.69 for caries experience and from 0.10 to 0.18 for active caries, indicating that familial caries aggregation has to be considered an important factor.

Caries experience varied in the eleven areas which are based on postal codes from 26.8 to 65.7% (*p* < 0.001). Active caries varied from 9.9 to 29.1% (*p* < 0.001). For both outcomes, the areas with the highest numbers as well as the area with the lowest number were the same, indicating a strong effect of the place of residence.

### Prediction of caries in younger siblings based on the caries status of first-born child

Table 3 shows the results of the comparison of children with lower birth order to first-born children, adjusted for potential confounding factors such as gender, age or nationality using mixed effect models. For caries experience as well as active carious lesions, the level of significance dropped or disappeared when models were adjusted for age (models II and III).

**Table 3:** Individual comparison of risks for caries experience (dmft + DMFT) or active carious lesions (dt + DT) in subsequently birth-ordered children to the first-born child. Risk comparisons are given as odds ratios (OR) with the respective 95% confidence intervals (CI 95%). Models are adjusted for birth order and gender (model I); for birth order, gender and age (model II); or for birth order, gender, age and nationality (model III)

Comparison of birth orders	Caries experience OR (CI 95%) *p *value			Active caries OR (CI 95%) *p *value		
	Model I	Model II	Model III	Model I	Model II	Model III
2nd to 1st	0.83 (0.72–0.97) *p* = 0.012	1.03 (0.86–1.23) *p* = 0.973	1.05 (0.88–1.26) *p* = 0.868	1.53 (1.27–1.84) *p* < 0.001	1.23 (0.99–1.52) *p* = 0.063	1.27 (1.02–1.57) *p* = 0.024
3rd to 1st	0.78 (0.58–1.06) *p* = 0.148	1.07 (0.77–1.50) *p* = 0.948	1.12 (0.80–1.55) *p* = 0.832	2.04 (1.44–2.88) *p* < 0.001	1.47 (1.00–2.15) *p* = 0.048	1.53 (1.05–2.24) *p* = 0.020
4th and later-born to 1st	0.81 (0.37–1.78) *p* = 0.903	1.24 (0.55–2.77) *p* = 0.905	1.32 (0.59–2.95) *p* = 0.801	2.04 (0.82–5.05) *p* = 0.182	1.32 (0.52–3.36) *p* = 0.872	1.42 (0.56–3.61) *p* = 0.767

To analyse effects within families, i.e. the intrafamilial effect, caries experience and active carious lesions in all subsequent siblings of a family were predicted based on the caries status of the first child. With odds ratios of 3.67 (95% CI 3.01–4.35, *p *value < 0.001) for caries experience and 3.48 (95% CI 2.57–4.72, *p *value < 0.001) for active carious lesions, an increased risk was present for younger siblings if the first-born child in the family already had caries.

Such associations were also noted when caries experience and active carious lesions in children of discrete birth orders were predicted based on the caries status of the first child, age, gender, nationality and age difference to the eldest child within the respective family (Table 4). This intrafamilial effect increased in children of higher birth order. Absolute age at time of examination had a strong influence on the outcomes. The larger the age difference between siblings, the smaller the caries risk for younger siblings. Although gender was not statistically significant, a trend towards a smaller risk was seen for lower birth orders and females. Nationality was statistically significant, especially for higher birth orders. This pattern was noticed for most factors, as with increasing birth order, statistical significance dropped or disappeared and the strongest influence was noticed for the second-born sibling.

**Table 4:** Prediction of caries experience (dmft + DMFT) and active carious lesions (dt + DT) in younger siblings based on the caries status of the first-born child within the respective family (intrafamilial), age of the respective child, age difference to the first-born child (age diff), gender and nationality. Shown are odds ratios (OR) with 95% confidence intervals (CI 95%) for the risks and corresponding *p *values. *n.a.* not applicable

Comparison of birth orders	Caries experience OR (CI 95%)* p *value					Active caries OR (CI 95%) *p *value				
	Intrafamilial	Age	Age diff	Gender	Nationality	Intrafamilial	Age	Age diff	Gender	Nationality
1st child	n.a	0.71 (0.58–0.87) *p* < 0.001	n.a	0.95 (0.82–1.10) *p* = 0.489	1.86 (1.60–2.16) *p* < 0.001	n.a	0.63 (0.48–0.83) *p* = 0.001	n.a	0.82 (0.68–1.00) *p* = 0.047	1.94 (1.60–2.35) *p* < 0.001
2nd to 1st	3.20 (2.74–3.75) *p* < 0.001	2.84 (2.26–3.57) *p* < 0.001	0.81 (0.740.88) *p* < 0.001	0.82 (0.71–0.96) *p* = 0.013	1.71 (1.46–2.01) *p* < 0.001	2.43 (1.97–3.00) *p* < 0.001	0.87 (0.67–1.13) *p* < 0.001	0.83 (0.76–0.91) *p* < 0.001	0.91 (0.76–1.08) *p* = 0.292	2.04 (1.71–2.44) *p* < 0.001
3rd to 1st	3.29 (2.22–4.86) *p* < 0.001	3.15 (1.81–5.50) *p* < 0.001	0.74 (0.56–0.98) *p* = 0.035	1.20 (0.81–1.77) *p* = 0.364	1.84 (1.23–2.74) *p* = 0.003	2.52 (1.51–4.20) *p* < 0.001	1.84 (0.99–3.41) *p* = 0.229	0.68 (0.50–0.91) *p* = 0.011	1.13 (0.73–1.74) 0.575	2.60 (1.69–3.99) *p* < 0.001
4th and later-born to 1st	4.22 (1.26–14.09) *p* = 0.019	8.75 (1.52–50.49) *p* = 0.096	0.47 (0.18–1.26) *p* = 0.134	0.58 (0.17–2.00) *p* = 0.392	2.94 (0.84–10.21) *p* = 0.090	3.47 (0.61–19.69) *p* = 0.161	5.42 (0.87–33.64) *p* = 0.271	0.30 (0.09–0.96) 0.042	0.46 (0.12–1.78) *p* = 0.259	1.78 (0.43–7.42) *p* = 0.428

## Discussion

In the canton of Basel-Stadt, parents can either refer their child to their private dentist or to the University Center for Dental Medicine Basel if an untreated carious lesion is detected during annual dental examination. The primary objective of the responsible authorities is, however, that all children should have a caries-free dentition. As a caries-free dentition was present in only approximately half of the examined schoolchildren, this study focused on possibilities to identify families with children in need of targeted dental interventions. To do so, the caries status of the first-born child was evaluated as a possible predictor for caries in younger siblings from the same family. The results of the present cross-sectional study in schoolchildren aged from 4 to 15 years could confirm our hypothesis. Younger siblings had up to a four-fold higher risk for caries experience and a two-to-three-fold higher risk for active carious lesions if the firstborn child already presented with carious lesions.

Caries was shown to be family dependent which is in accordance with other studies [12, 19–21]. In a Norwegian study, the correlation between siblings aged 6 to 18 in caries was analysed. A strong family effect, with a between-family variability in the range of 13–29% was noted [19]. In a recent Danish study comparing 15-year-olds and their biological siblings born within three calendar years, a substantial familial aggregation of caries was described, with caries in the corresponding sibling as the most important caries predictor [12]. All these studies analysed different age groups, and age is known to be an important risk factor for caries. However, recent studies indicate that there is an increasing shift from children to adults [2, 16]. For the authorities in the canton of Basel-Stadt, however, the goal is to have a caries-free dentition in every child living in the canton independent of its age.

The assessment of factors influencing oral health is a significant component of dental public health research. Prevention programs should focus on the specific risk groups for caries. There is an association between socioeconomic factors, which usually are shared by family members living in the same household, and dental caries [4, 10]. This association appears to be stronger in developed countries such as Switzerland [10]. There are ethical boundaries in Switzerland to access certain socioeconomic factors such as employment, education or income in each family, whereas the caries status of the first-born child can be used as a potential indicator to detect vulnerable families. In the present study, two other influencing factors usually shared by families, namely nationality and the place of residence, were also associated with caries. Schoolchildren with a migrant background are a caries-risk group. These findings corroborate earlier studies carried out in Switzerland [6–8].

Geomapping, i.e. the geographical mapping of health data, has been described as a new tool to monitor health risk for medical as well as dental health purposes [22–24]. In Sweden, this tool has been used not only to illustrate the inequalities in caries risk among children living in Halland region in southwest Sweden but also to evaluate the effects of allocating more money to parishes inhabited by children with increased caries risk [23–25]. Geographical variations in caries risk were also noticed in the present study. Including geographical data may therefore be another instrument to refine and improve the assessment of vulnerable families in the canton of Basel-Stadt.

There has been an increased interest in the effect of birth order on various aspects of child health such as risk-taking behaviour, child maltreatment or childhood thinness [26–28]. Likewise, recent studies focused on the influence of birth order on dental caries in children [14, 15]. In a Norwegian study in 5-year-old children, caries experience was not associated with the presence of older siblings in the family [15]. This is in accordance with the present study, where later-born children did not have an increased risk for caries when compared to their eldest sibling. In contrast, Julihn et al. (2020) reported a statistically significant positive association between birth order and caries development in Swedish children between age 3 and 7 years, with younger siblings having a significantly increased risk of developing new caries lesions compared with first-born children. In this study, however, the intrafamilial correlation was not included in the analyses [14]. These Scandinavian studies were all carried out in young children with deciduous teeth, while schoolchildren aged 4 to 18 were included in the present study. Age and the difference in age between siblings are expected to have a strong influence on the outcomes. There are also differences in study design. This study and the study of Wigen et al. (2011) have a cross-sectional design, whereas Julihn et al. (2020) analysed dental caries development over time, i.e. longitudinally. By focusing on young children and analysing increments in caries over time, it seems to be possible to identify families in need of oral healthcare at an early stage.

All schoolchildren living in the canton of Basel-Stadt are examined annually and, therefore, constitute the study sample this register-based study. The dental examinations are performed by specialised dentists of the University Center for Dental Medicine Basel. Although the dentists are not regularly calibrated, their differences in identifying caries were small as shown by the 50% IQR values that ranged from 0.47 to 0.53 for caries experience. However, an increased accuracy might be achieved by a yearly calibration.

Despite substantial scientific progress in understanding the pathogenesis of oral diseases and their contributing factors over the last decades, untreated dental caries remains one of the most prevalent health conditions worldwide [2, 4]. In high-income countries such as Switzerland, interventions focusing on highly technical aspects of dentistry are getting increasingly criticised as these approaches are not attempting to address existing inequalities in oral health [4, 5]. It has been recommended that dental services should become more integrated in the wider healthcare system and thereby be more accessible and responsive to the oral health needs of the respective population [5]. This is also the aim of the responsible authorities in the canton of Basel-Stadt, as mandatory dental examinations for all schoolchildren exist. However, a more focused and structured approach may help to further identify vulnerable families in need of caries treatment.

As the null hypothesis was rejected, this study presents evidence that the caries status of the eldest sibling of a family may serve as a predictor for caries in younger children from the same family even when adjusted for known confounding factors such as age, age difference and nationality. Combined with other factors such as nationality and the family’s place of residence, this finding can help to orient prevention measures in a more targeted manner to children in need.
